# The Genomic Makeup of Nine Horse Populations Sampled in the Netherlands

**DOI:** 10.3390/genes10060480

**Published:** 2019-06-25

**Authors:** Anouk Schurink, Merina Shrestha, Susanne Eriksson, Mirte Bosse, Henk Bovenhuis, Willem Back, Anna M. Johansson, Bart J. Ducro

**Affiliations:** 1 Centre for Genetic Resources, the Netherlands (CGN), Wageningen University & Research, P.O. Box 338, 6700 AH Wageningen, The Netherlands; 2 Animal Breeding and Genomics, Wageningen University & Research, P.O. Box 338, 6700 AH Wageningen, The Netherlands; merina44_sta@yahoo.com (M.S.); mirte.bosse@wur.nl (M.B.); henk.bovenhuis@wur.nl (H.B.); bart.ducro@wur.nl (B.J.D.); 3 Department of Animal Breeding and Genetics, Swedish University of Agricultural Sciences, P.O. Box 7023, 75007 Uppsala, Sweden; Susanne.Eriksson@slu.se (S.E.); anna.johansson@slu.se (A.M.J.); 4 Department of Equine Sciences, Faculty of Veterinary Medicine, Utrecht University, Yalelaan 112-114, 3584 CM Utrecht, The Netherlands; W.Back@uu.nl; 5 Department of Surgery and Anaesthesiology of Domestic Animals, Faculty of Veterinary Medicine, Ghent University, Salisburylaan 133, B-9820 Merelbeke, Belgium

**Keywords:** genetic diversity, horses, inbreeding, population structure, relatedness, runs of homozygosity

## Abstract

The spectrum of modern horse populations encompasses populations with a long history of development in isolation and relatively recently formed types. To increase our understanding of the evolutionary history and provide information on how to optimally conserve or improve these populations with varying development and background for the future, we analyzed genotype data of 184 horses from 9 Dutch or common horse populations in the Netherlands: The Belgian draft horse, Friesian horse, Shetland pony, Icelandic horse, Gelder horse, Groninger horse, harness horse, KWPN sport horse and the Lipizzaner horse population. Various parameters were estimated (e.g., runs of homozygosity and F_ST_ values) to gain insight into genetic diversity and relationships within and among these populations. The identified genomic makeup and quantified relationships did mostly conform to the development of these populations as well as past and current breeding practices. In general, populations that allow gene-flow showed less inbreeding and homozygosity. Also, recent bottlenecks (e.g., related to high selective pressure) caused a larger contribution of long ROHs to inbreeding. Maintaining genetic diversity through tailor-made breeding practices is crucial for a healthy continuation of the investigated, mostly inbred and (effectively) small sized horse populations, of which several already experience inbreeding related issues.

## 1. Introduction

In horses, genetic diversity is predominantly observed between breeds or types, where little variation is present within breeds [1]. The large variety in horse breeds and populations corresponds to a variety in purposes of the horse to mankind. The horse probably has the greatest diversity in purposes of all domesticated animal species and has had a large impact on the civilization of humans. Horses were a food source and later used for transportation, draft power, agricultural work, hunting and warfare [2]. Most of these historical purposes are still performed by horses in some part of the world [3]. In addition to these different purposes, the evolution from the domesticated founding horses into the modern breeds was also driven by geographic and environmental (climatic) conditions. Geographic isolation and harsh conditions were the driving forces behind the development of the Icelandic horse from its ancestors centuries ago, that were brought to Iceland from the Shetland Isles and Nordic countries [4]. Shetland ponies developed in isolation on the Shetland Isles, and their characteristic type was further shaped after being exported to the UK for use in mines where the smallest and strongest ponies were preferred [4,5].

The majority of the breeds, however, developed under less isolated conditions. A certain exchange of genetic material between breeds took place in the past, although predominantly breeding of horses that looked alike was practiced. In the late 19th century studbooks emerged, formalizing breeding goals and breeding standards, by which the voluntary exchange of genetic material between breeds decreased. This initiated that some breeds became closed, prohibiting the introduction of genetic material from other breeds [6].

With increasing mechanization most of the original purposes for keeping horses became less important and new breeding goals related to sports, companionship as well as general leisure activities emerged [2]. As a consequence, breeding goals often became narrower and maintenance of specific breed characteristics were prioritized, such as conformation, color, or specific gaits. These phenomena can be exemplified by the recent history of the Dutch warmblood horses [7]. Warmblood horses were used for transportation (riding and harnessing) and for agricultural work in the field [7]. The increased focus on sports performance in competition (dressage and show jumping) brought its substantial improvement of athletic abilities. The original versatile warmblood type is still present and nowadays called Gelder horse [7]. Next to the sport horses, a second type emerged from the Gelder horse, which is bred for harnessing and called harness horse. Harnessing in the Netherlands is a show sport where the performance and thus the conformation of the horse plays an important role. In only a few generations, distinct breed types have thus been developed within the warmblood horse population by specialized breeding goals and introgressing specific features from other breeds (e.g. spectacular knee action in the harness horse from Hackney and American Saddlebred horse populations). Divergence obtained in only a few generations often reduces genetic diversity within breeds or populations as a result of the extensive use of a few popular sires that comply with the breeding goal, e.g., [8,9].

The range of modern horse breeds varies from breeds with development in isolation for quite some time to relatively recently formed breeds or types as can be seen in the Dutch warmblood horse population. To comprehend the evolutionary history of various horse populations and to inform about how to best conserve or improve horse populations with varying development and background for the future, we quantified genetic diversity and relationships within and among horses in the Netherlands using genome-wide SNP genotype data. The 9 horse populations that were sampled, represent almost all native Dutch horse populations and the largest horse populations in the Netherlands. Icelandic horses and Lipizzaner horses were included as non-native and less common populations, representing breeds with a longer history of organized breeding and development. We expected that the identified genomic makeup of these populations conforms to the development of the population (as far as we know) as well as past and current breeding practices. Prior studies have investigated genetic diversity for a few of these populations but based only on pedigree information [8,9,10] or short tandem repeat loci as genetic markers [11]. To our best knowledge, this is the first study of its kind for the populations that were sampled in the Netherlands.

The identified genomic makeup and quantified relationships did mostly conform to the development of the populations (as far as we know) as well as past and current breeding practices. Maintaining genetic diversity through tailor-made breeding practices is crucial for a healthy continuation of the investigated, mostly inbred and (effectively) small sized horse populations, of which several already experience inbreeding related issues.

## 2. Materials and Methods

### 2.1. Horses and Data Collection

Data were available on 184 horses from 9 populations (Table 1). Belgian draft horses, Gelder horses, Groninger horses, harness horses, KWPN sport horses and Lipizzaner horses were sampled with a focus on our genetic diversity research. Samples were collected by researchers from Utrecht University with help from various studbooks in the Netherlands. Within each population, relationships among horses were reduced as much as possible to represent the diversity present within a population. Besides breed classification, no additional information was available. For Friesian horses, Shetland ponies and Icelandic horses (born in the Netherlands) samples were originally gathered to perform genome-wide association studies for diseases and contained close relatives (e.g., several full-sibs, a reasonable number of half-sibs, and parents and offspring). Among those samples we selected horses and ponies for our genetic diversity research that did not share parents based on pedigree information. All samples were collected with the horse owner’s consent. Moreover, blood sample collection from Shetland ponies and Icelandic horses was approved by the Board on Animal Ethics and Experiments from Wageningen University (experiments 2009005 and 2010109).

Almost all native Dutch horse populations [12] were included in our study (warmblood: Gelder horse, harness horse, KWPN sport horse and Groninger horse; coldblood: Friesian horse and Belgian draft horse (although of Belgian origin, ample exchange of horses between the Belgian and Dutch population occurs, whereby it can be considered as one population today [13])). The Shetland pony population was included as it is one of the largest horse populations in the Netherlands (besides the KWPN sport horse and Friesian horse populations [14]). The Icelandic horse and Lipizzaner horse populations were included to represent horse populations with long history of organized breeding, and because of their potential relatedness to the Shetland pony and Dutch warmblood horse populations, respectively. The horse populations included in our study allowed us to investigate and compare almost all native Dutch horse populations, several of the largest horse populations present in the Netherlands, and populations with varying development and background (e.g., older versus more recently formed breeds).

### 2.2. Genotypes and Quality Control

Friesian horses were genotyped using the Illumina^®^ EquineSNP50 Genotyping BeadChip containing 54,602 SNPs, whereas Lipizzaner horses and Belgian draft horses were genotyped using the Illumina^®^ equine HD array containing 65,157 SNPs. In each of the other populations, some horses were genotyped using the BeadChip and other horses were genotyped using the HD array. Genotypes are available upon reasonable request. Only SNPs that were present on both arrays (*n* = 45,986) were used in our study.

Quality control was performed using PLINK software (version 1.9) [15,16]. SNPs present on the X chromosome were excluded. Furthermore, SNPs with a call-rate < 90% were removed. 43,779 SNPs remained after quality control. No horses were removed, as genotyping rate was > 90% for all horses.

### 2.3. Parameters Within Populations

#### 2.3.1. Inbreeding Coefficient

Inbreeding coefficients were estimated to study the genetic diversity status within the populations. SNPs in linkage disequilibrium (LD) can reduce the informativeness of a dataset [17] and may lead to a less accurate estimation of inbreeding coefficients. Therefore, to be able to accurately estimate inbreeding coefficients, LD-based SNP pruning was performed using the *indep-pairwise* command in PLINK software (version 1.9) [15,16]. Pruning was performed using a window of 100 SNPs that shifted 25 SNPs at a time. SNPs within a window were removed when the pairwise LD was ≥ 0.1. After the LD-based pruning, 8704 SNPs remained. Subsequently, inbreeding coefficients ($f_{i}$) were estimated taking into account all horses using the *het* command in PLINK (version 1.9) [15,16], where

(1) $$f_{i}=\frac{\left(O_{i}-E_{i}\right)}{\left(L_{i}-E_{i}\right)},$$

and

(2) $$E_{i}=\sum_{j=1}^{L_{i}}1-2p_{j}\left(1-p_{j}\right),$$

$O_{i}$ is the observed number of homozygous SNPs for individual i, and $E_{i}$ is the expected number of homozygous SNPs based on genotype data from all horses. $L_{i}$ is the number of genotyped SNPs, and $p_{j}$ is the major allele frequency of a SNP at locus j.

Inbreeding results in homozygosity. Another way of estimating inbreeding coefficient is therefore to determine the proportion of the autosomal genome that is homozygous. Runs of homozygosity (ROHs), long stretches of homozygous SNPs, were identified (see Section 2.3.4) and used to estimate inbreeding coefficients ($f_{i,ROH}$) as defined by McQuillan and colleagues [18]:

(3) $$f_{i,ROH}=\frac{\sum L_{i,ROH}}{L_{AUTOSOME}}$$

where $\sum L_{i,ROH}$ is the sum of the lengths of the ROHs for individual i and $L_{AUTOSOME}$ is the length of the autosomal equine genome covered by the SNP array, in this case 2,242,939,370 bp.

#### 2.3.2. Linkage Disequilibrium

We determined the extent and diminishing of LD within each population, as among other things it shapes haplotypes, is related to the effective population size and is of importance in association studies. LD between all SNPs on a randomly chosen chromosome (ECA2) using genotype data was calculated with the *r2* command in PLINK software (version 1.9) [15,16]. To determine the diminishing of LD with increasing physical distance between SNPs, mean LD was expressed as a function of distance. For that purpose, pairs of SNPs were separated into bins of 10 kb in size according to the intermarker distance. Subsequently, mean LD and distance was calculated per (non-overlapping) bin of 10 kb.

#### 2.3.3. Pairwise Identity-by-Descent

Pairwise identity-by-descent (IBD) was calculated to quantify and visualize relationships between horses within a population. Pairwise IBD was estimated using the *genome* command in PLINK (version 1.9) [15,16] and the dataset pruned for LD. A method-of-moments approach was applied as described by Purcell and colleagues [16] using allele frequencies and identity-by-state to estimate pairwise IBD estimated as the proportion of the genome being IBD:

(4) $$\hat{\pi }=P(IBD=2)+\frac{1}{2}\times P(IBD=1)$$

#### 2.3.4. Runs of Homozygosity

Runs of homozygosity (ROHs), long stretches of homozygous SNPs, in the genome were identified to increase our understanding of the genetic diversity status of the investigated horse populations and to be able to speculate about their evolutionary history. ROHs were identified using the *homozyg* command in PLINK (version 1.9) [15,16] that applies a sliding window approach. A window of 50 SNPs (*homozyg-window-snp*) was slid across the genome, one SNP at a time. At each window position it was determined whether the window was homozygous. We allowed the window to contain ≤ 2 missing genotype calls (*homozyg-window-missing*) but no heterozygous genotypes (*homozyg-window-het*). Each SNP was present in about 50 windows. For each SNP, the percentage of windows being homozygous was calculated, and when this percentage was ≥ 5% (*homozyg-window-threshold*), that particular SNP was defined to be present in a homozygous segment. A segment had to contain ≥ 50 SNPs (*homozyg-snp*), had to be ≥ 1 Mb in length (*homozyg-kb*), and had to contain at least 1 SNP per 5000 kb (*homozyg-density*) to be called an ROH. Also, the distance between two adjacent SNPs had to be ≤ 5000 kb (*homozyg-gap*).

### 2.4. Relationships Among Populations

#### 2.4.1. Multidimensional Scaling

To examine genetic relationships within and between the populations, genome-wide identity-by-state distances between the 184 horses were calculated using the *cluster* command in PLINK software (version 1.9) [15,16]. The genetic distances were visualized in a multidimensional scaling (MDS) plot that represented the first two components identified with the *mds-plot* command in PLINK software (version 1.9) [15,16]. The MDS plot also served as a quality control, as it exposes potential breed-misclassification of a horse.

#### 2.4.2. F_ST_ Approach

To get a quantitative measure of genetic differentiation between the horse populations, fixation index F_ST_ was estimated using the *fst* command in PLINK (version 1.9) [15,16] and the dataset pruned for LD. F_ST_ values per SNP were estimated based on the method described by Weir and Cockerham [19]. A weighted average of F_ST_ values per SNP between two populations was estimated to establish the genetic differentiation between these populations.

#### 2.4.3. Admixture Analysis

An admixture analysis for K = 1 to 15 was performed on the dataset pruned for LD using the software ADMIXTURE (version 1.3.0) [20]. Five EM steps were performed to prime the main algorithm and cross-validation was done for each K-value to explore the optimal number of clusters present within the dataset.

#### 2.4.4. Phylogenetic Tree

A distance matrix (*distance-matrix*) was constructed in PLINK (version 1.9) [15,16] using the dataset pruned for LD. Using this matrix, a neighbor-joining tree was created in PHYLIP [21] and depicted in FigTree (http://tree.bio.ed.ac.uk/software/figtree/).

## 3. Results

### 3.1. Parameters Within Populations

#### 3.1.1. Inbreeding Coefficient

**Figure A1:**
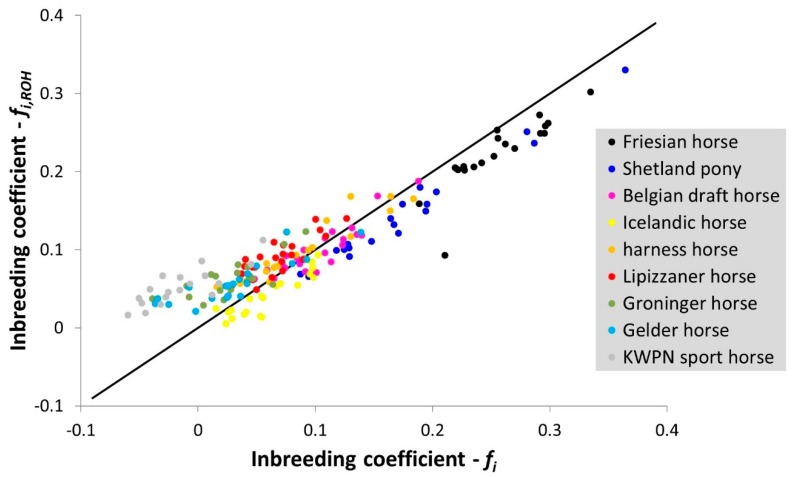
The correlation between inbreeding coefficients estimated using expected and observed homozygosity ($f_{i}$) and based on runs of homozygosity ($f_{i,ROH}$ ) using a window of 50 SNPs in size. The black solid line represents the case where $f_{i}$ = $f_{i,ROH}$.

The mean inbreeding coefficient for all horses was 8.8% (Table 2). The highest mean inbreeding coefficients were observed in the Friesian horse and Shetland pony populations. The lowest mean inbreeding coefficients were observed in the KWPN sport horse and Groninger horse populations (Table 2). However, the lowest mean inbreeding coefficient based on ROHs was observed in the Icelandic horse population (Table 2). For several horses, the individual inbreeding coefficient exceeded 25% (Figure A1), which equals the expected value from a full-sib mating.

**Figure A2:**
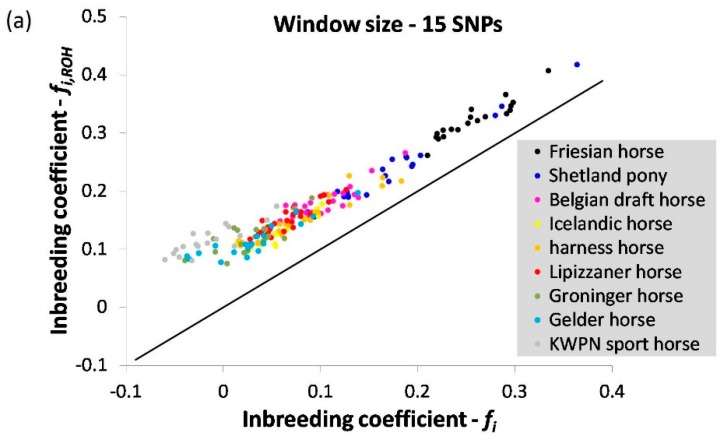

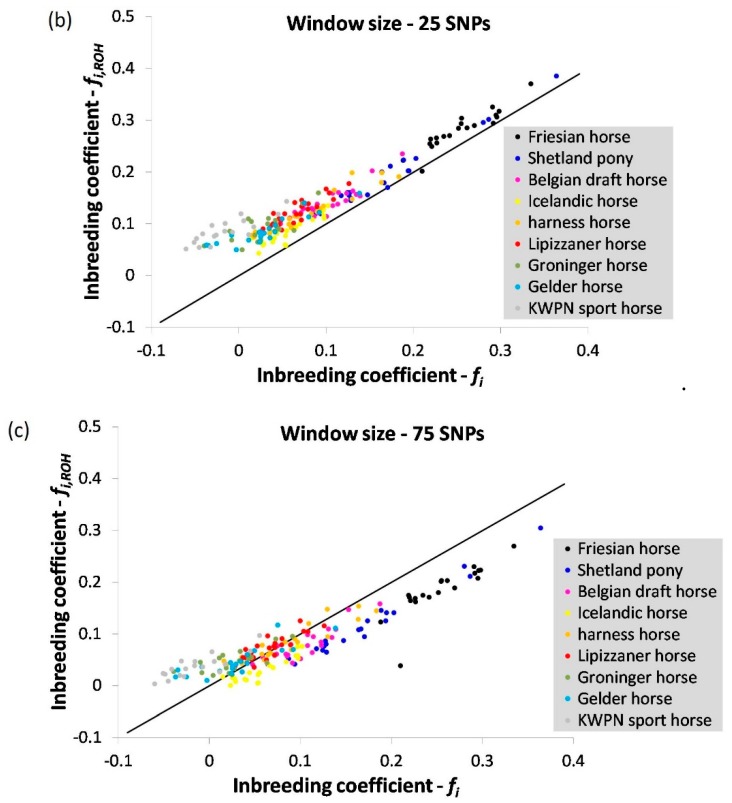
The correlation between inbreeding coefficients estimated using expected and observed homozygosity ($f_{i}$) and based on runs of homozygosity ($f_{i,ROH}$) using a window of (**a**) 15, (**b**) 25 and (**c**) 75 SNPs in size. The black solid line represents the case where $f_{i}$ = $f_{i,ROH}$.

Inbreeding coefficients estimated using expected and observed homozygosity ($f_{i}$) and based on ROHs ($f_{i,ROH}$) were most similar when a window size of 50 was applied (Figure A1 and Figure A2). As expected, the estimated inbreeding coefficient $f_{i,ROH}$ decreased when a larger window size was considered (Figure A2, Table A1). When a window or segment did not pass the criteria set (e.g., containing 3 or more missing genotype calls within a window), homozygous segments were disregarded despite their contribution to homozygosity in the genome, underestimating true genomic inbreeding.

Interesting to notice (Figure A1) is that the inbreeding coefficient based on ROHs ($f_{i,ROH}$) in the warmblood populations often was higher compared to their inbreeding coefficient based on expected and observed homozygosity ($f_{i}$). Ascertainment bias might contribute to this observation, as especially warmblood populations contributed to the development of the SNP arrays. Heterozygous SNPs within populations used to develop these arrays are more likely to be selected. These arrays therefore contain a biased set of pre-ascertained SNPs likely to underestimate homozygosity within the populations used to develop the arrays.

#### 3.1.2. Linkage Disequilibrium

**Figure 1:**
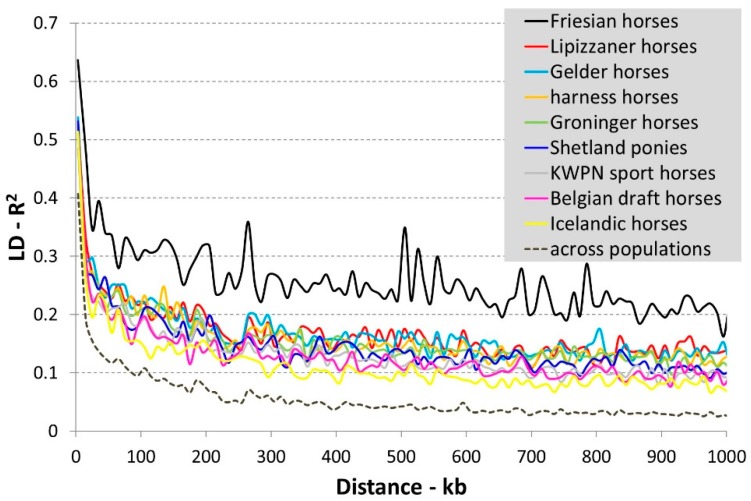
Diminishing of linkage disequilibrium (LD) with increasing physical distance between SNPs across and within the investigated horse populations. LD expressed as a function of distance based on genotype data from a randomly chosen chromosome (ECA2). Mean LD and distance was calculated per (non-overlapping) bin of 10 kb.

The diminishing of LD with increasing physical distance between SNPs across and within the investigated horse populations is presented in Figure 1. Within populations, highest LD was observed in the Friesian horse population and lowest LD in the Icelandic horse population (Figure 1). On average longer haplotypes and a smaller effective population size are to be expected especially in the Friesian horse population compared to the Icelandic horse population.

#### 3.1.3. Pairwise IBD

**Figure A3:**
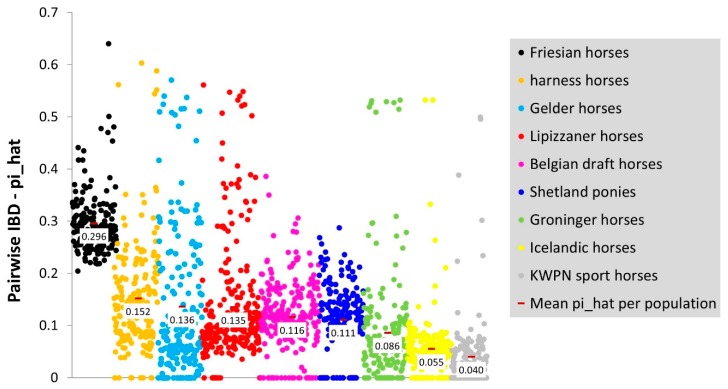
Pairwise identity-by-descent (IBD) within each population, ordered from highest to lowest mean $\hat{\pi }$ per population (where $\hat{\pi }=P(IBD=2)+\frac{1}{2}\times P(IBD=1)$).

Mean pairwise IBD was highest in the Friesian horse population (0.296) and lowest in the KWPN sport horse population (0.040; Figure A3). Reasonable variation was observed in the proportion of the genome being IBD within the Gelder horse, Groninger horse, harness horse and Lipizzaner horse population (Figure A3). Despite the attempt to reduce relationships among horses within a population as much as possible, several (close) relatives seem present.

#### 3.1.4. Runs of Homozygosity

A total of 6170 ROHs were identified in the 9 horse populations (Table 2). Almost 25% of these ROHs were found in the Friesian horse population. The number of ROHs identified per horse ranged from 5 to 89 (Table 2). Mean length of an ROH was 6.5 Mb (Table 2). Interestingly, the longest ROH observed in a horse—a Shetland pony—was 91.6 Mb in length (= 49.3% of chromosome 1).

**Figure 2:**
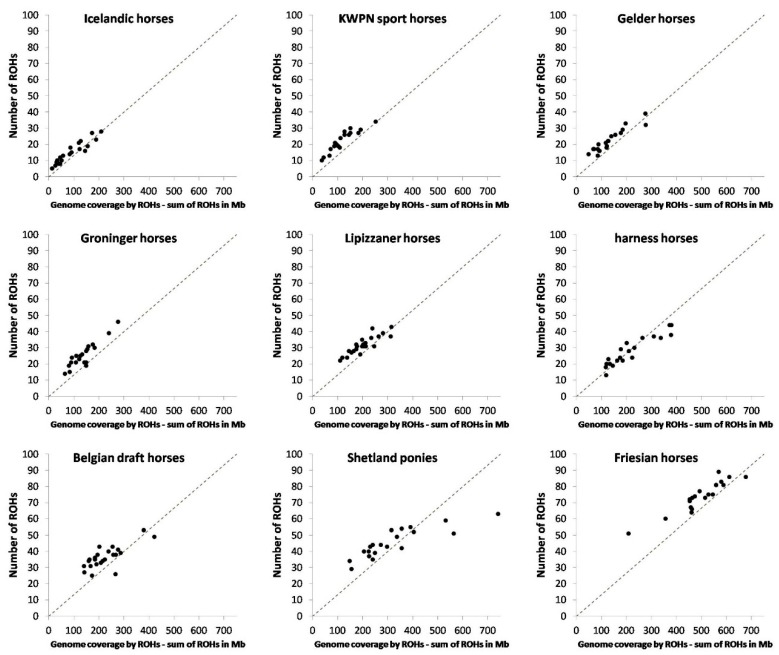
Number of ROHs versus genome coverage by ROHs (as sum of ROHs in Mb) per population. Genome coverage, being the inbreeding coefficient based on ROHs, was calculated as $f_{i,ROH}=\frac{\sum L_{i,ROH}}{L_{AUTOSOME}}$. Populations are ordered according to average inbreeding coefficient within population (${\bar{f}}_{i,ROH}$), from lowest to highest.

**Figure A4:**
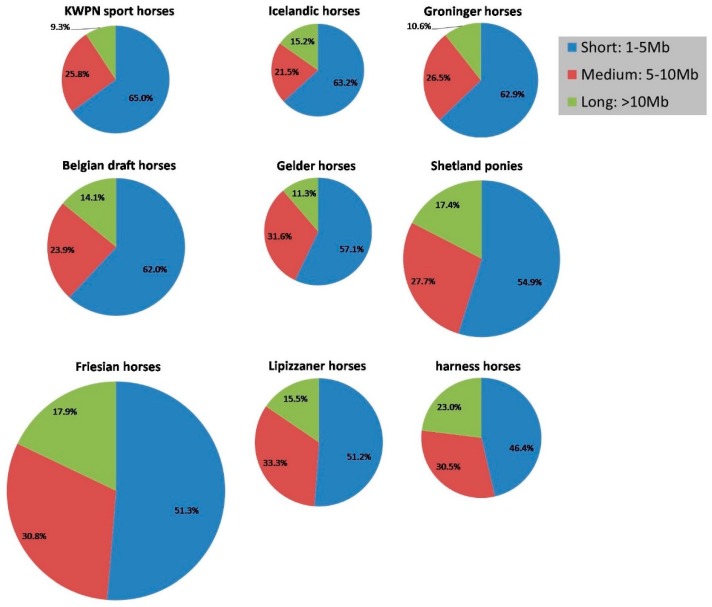
Distribution of size of ROHs per population, where short ROHs were 1 to 5 Mb in size, medium ROHs were 5 to 10 Mb in size and long ROHs were >10 Mb in size. Populations are ordered from having the highest percentage of short ROHs to having the lowest percentage of short ROHs. For example, within the KWPN sport horse population 400 ROHs were identified of which 260 were short (= 65%). The size of the pie chart reflects the average number of ROHs within a population.

The number and length of ROHs in the genome varied between horses from different populations (Table 2, Figure 2 and Figure A4). The genome of Icelandic horses contained the fewest ROHs (on average 15.1) whereas the genome of Friesian horses contained the most ROHs (on average 74.3) (Table 2, Figure 2). On average shortest ROHs were identified in KWPN sport horses (5.4 Mb in length), whereas on average longest ROHs were identified in harness horses (7.8 Mb in length; Table 2). Percentage of long ROHs present within a population was highest for the harness horse population (23.0%) and lowest for the KWPN sport horse population (9.3%; Figure A4). Also, within the Shetland pony and Friesian horse populations a large part of the ROHs were long (respectively 17.4 and 17.9%).

**Figure 3:**
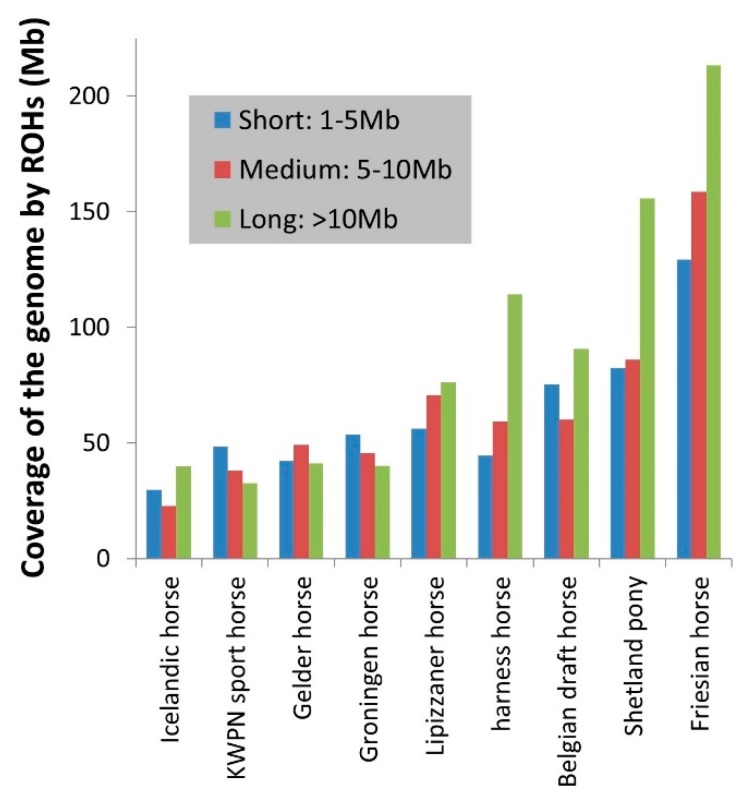
The average total size of the genome (in Mb) that is covered by short, medium and long ROHs per horse within a population. E.g. 114 Mb of the genome of a harness horse was on average covered by long ROHs (>10 Mb per ROH).

Homozygosity within the genome, that is inbreeding based on ROHs, varied considerably between the populations (Table 2, Figure 2 and Figure 3), from 0.6% in an Icelandic horse to 33.1% in a Shetland pony. Long ROHs contributed considerably to homozygosity in the genome of Icelandic horses, harness horses, Belgian draft horses, Shetland ponies and Friesian horses (Figure 3). In contrast, a substantial part of homozygosity in the genome of KWPN sport horses and Groninger horses originated from short ROHs (Figure 3).

### 3.2. Relationships Among Populations

#### 3.2.1. Multidimensional Scaling

**Figure 4:**
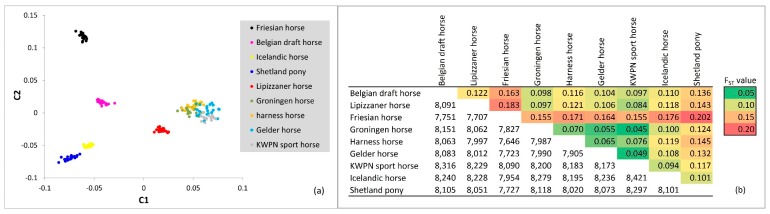
Visualization and quantification of relationships among the populations on the dataset pruned for LD: (**a**) multidimensional scaling plot of 184 horses from 9 populations sampled in the Netherland. The X-axis represents the first component (C1) and Y-axis the second component (C2) as calculated with the *cluster* and *mds-plot* commands in PLINK software (version 1.9) [15,16]; (**b**) mean F_ST_ values (above diagonal) and number of SNPs (below diagonal) on which the calculations between the populations are based.

The genetic relationships within and between the populations were visualized in an MDS plot (Figure 4a). In total 8.8% of the variance was explained by the first component, separating the warmblood and coldblood populations. The second component explained 7.2% of the variance present in the data and separated the pony-like (Icelandic horse and Shetland pony) and Friesian horse populations from the warmblood and Belgian draft horse populations. The first 4 components explained 25.6% of the total variance present. The Dutch warmblood populations clustered together (Figure 4a), whereas most other populations showed distinct clusters. None of the samples analyzed, seemed misclassified for breed.

**Figure A5:**
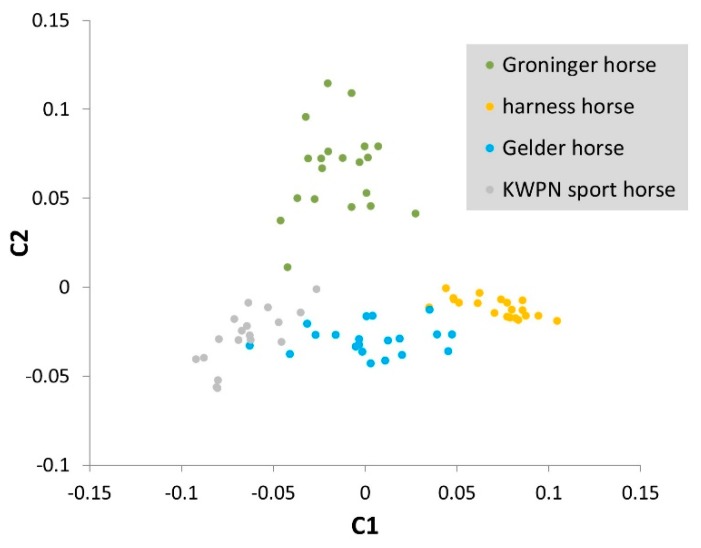
Multidimensional scaling plot constructed with genotype data from only the Dutch warmblood populations (Gelder horses, harness horses, KWPN sport horses and Groninger horses). The X-axis represents the first component (C1) and Y-axis the second component (C2) as calculated with the *cluster* and *mds-plot* commands in PLINK software (version 1.9) [15,16].

As genetic distances between the Dutch warmblood populations were small, a separate MDS plot was constructed with only data from these populations (Gelder horses, harness horses, KWPN sport horses and Groninger horses; Figure A5). In total 8.8% of the variance was explained by the first component and separated the KWPN sport horses and the harness horses from the cluster of Gelder and Groninger horses (Figure A5). The second component explained 5.6% of the variance present in the data and separated the Groninger horses from the KWPN sport horses, Gelder horses and harness horses.

#### 3.2.2. F_ST_ Approach

Based on estimated F_ST_ values, moderate genetic differentiation (F_ST_ values between 0.05 and 0.15; [22] in [23]) was observed between many of the populations (Figure 4b). Estimated genetic differentiation was greatest between the Friesian horse population and all other populations. Also, moderate genetic differentiation was observed between the Shetland pony population and all other populations. Again, genetic differentiation was limited between the four Dutch warmblood horse populations: the Gelder horses, harness horses, KWPN sport horses and Groninger horses.

#### 3.2.3. Admixture Analysis

**Figure 5:**
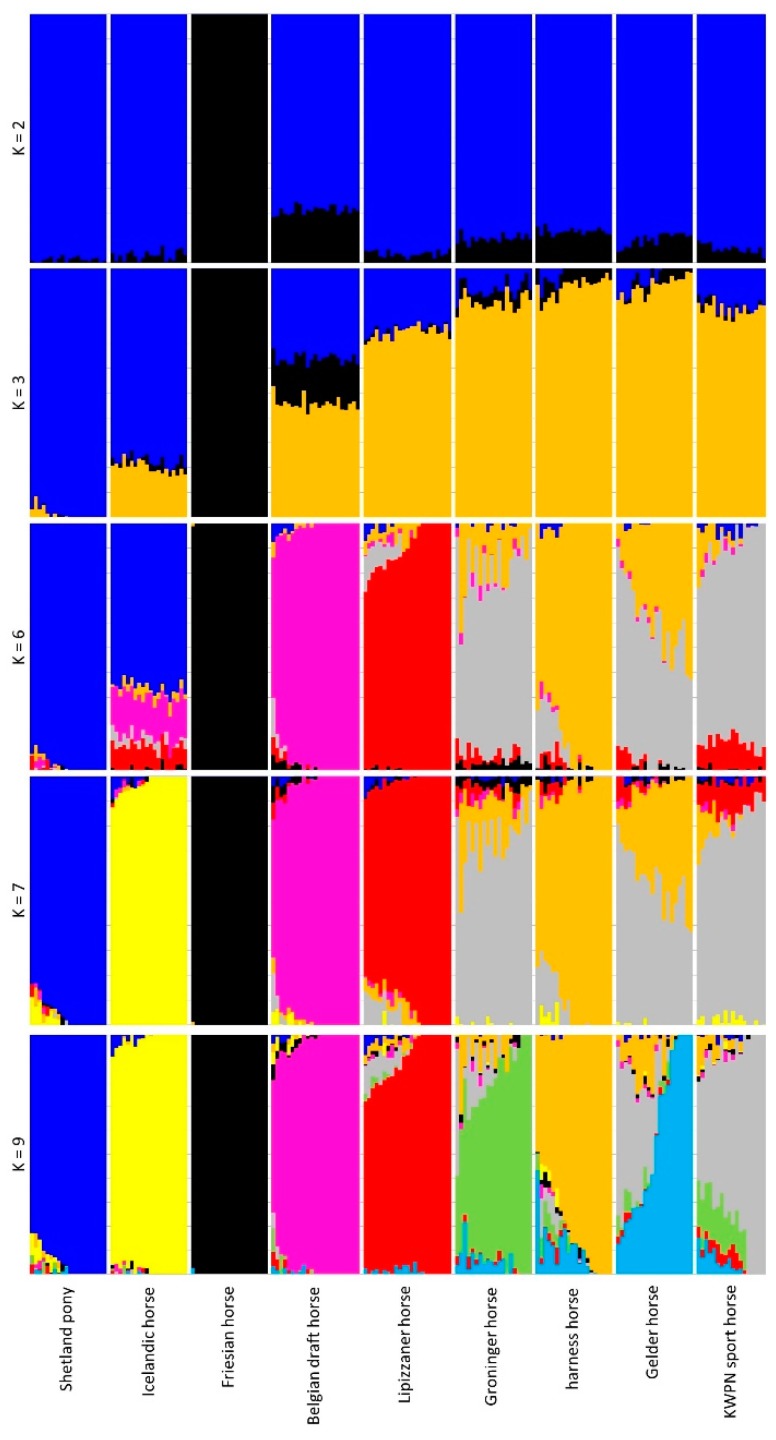
Inferred population structure for K = 2, 3, 6, 7 and 9 based on data of the sampled horses from 9 populations. The proportion of genome that is assigned to a particular cluster is represented by a particular color, where the proportion of the genome is shown on the Y-axis (0 to 1) and each vertical bar represents an individual horse on the X-axis.

**Figure A6:**
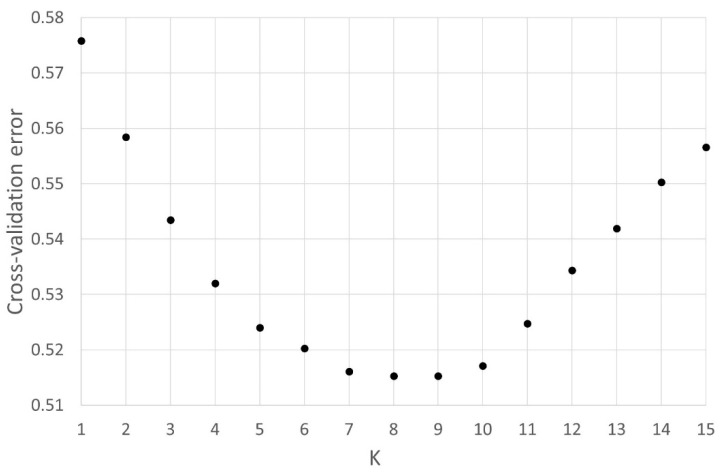
Lowest cross-validation error (0.5126) was found for K = 9 populations, the amount of populations investigated in this study.

The inferred population structure for K = 2 separated the Friesian horse population from the other populations (Figure 5). Furthermore, K = 3 separated the pony-like populations (Shetland ponies and Icelandic horses) and the warmblood horse populations (Lipizzaner, Groninger, harness and Gelder horse populations; Figure 5). The lowest cross-validation error (0.5126) was found for K = 9 clusters, the number of populations that were investigated, although the cross-validation error was only slightly higher for K = 8 (0.5153) and K = 7 (0.5161) (Figure A6).

**Figure A7:**
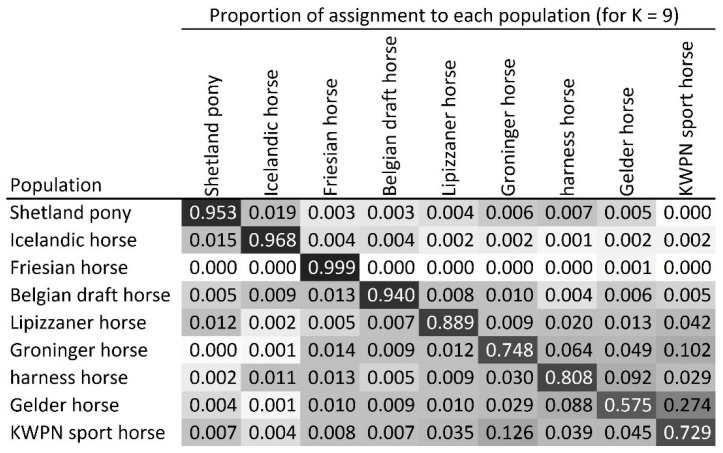
Average proportion of assignment to each population based on the admixture analysis and K = 9 clusters, the total number of populations investigated in the study and with the lowest cross-validation error (Figure A6).

Admixture in the Dutch warmblood populations (Groninger, harness and Gelder horse populations) was clearly visible (K = 9, Figure 5). Average proportion of assignment of the Dutch warmblood horses to their own population was only 0.575 for Gelder horses, 0.729 for KWPN sport horses, 0.748 for Groninger horses and 0.808 for harness horses (Figure A7). Average proportion of assignment of these populations to other populations was largely within the Dutch warmblood horse populations (e.g., for Gelder horses, the average proportion of assignment to the KWPN sport horse population was 0.274; Figure A7).

#### 3.2.4. Phylogenetic Tree

**Figure 6:**
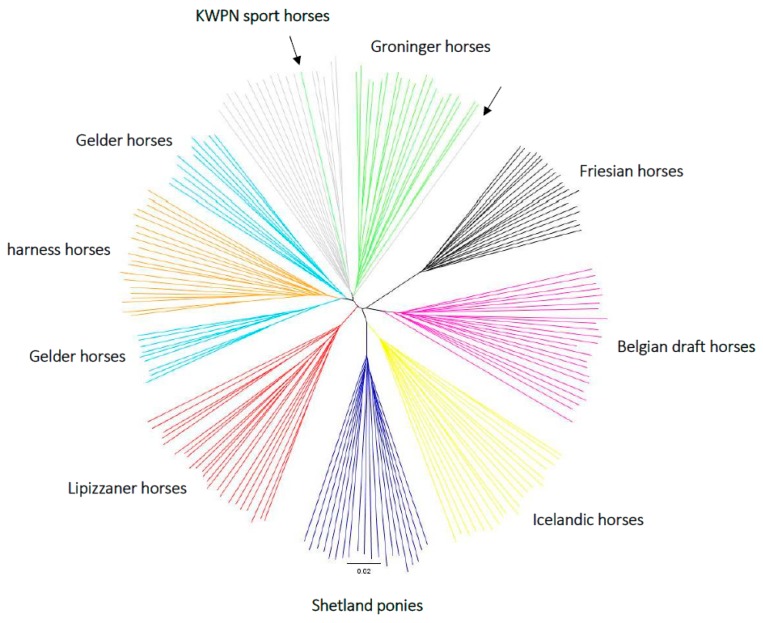
A neighbor-joining tree based on a distance matrix constructed in PLINK (version 1.9) [15,16] using genotype data of 184 horses from 9 populations sampled in the Netherlands. Each color represents a population: black = Friesian horse population, pink = Belgian draft horse population, yellow = Icelandic horse population, dark blue = Shetland pony population, red = Lipizzaner horse population, light blue = Gelder horse population, orange = harness horse population, grey = KWPN sport horse population and green = Groninger horse population. The arrows indicate the Groninger horse within the KWPN sport horse population clade and the KWPN sport horse within the Groninger horse population clade.

Almost all horses clustered with their own population (Figure 6). The Friesian horse population was most distant from the other populations. The Belgian draft and Lippizaner horse populations both had a distinct cluster. The pony-like populations (Shetland ponies and Icelandic horses) shared a clade, although the distance between these populations was larger compared to the distance between the Dutch warmblood populations. One Groninger horse clustered with the KWPN sport horse population, and one KWPN sport horse clustered with the Groninger horse population. These samples might have been switched, or represent truly admixed warmblood horses as the admixture analysis (Figure 5) assigned 41.5% to the KWPN sport horse population, 19.4% to the Groninger horse population, 16.3% to the harness horse population and 15.2% to the Gelder horse population for the KWPN sport horse that clustered with the Groninger horse population in the construction of the phylogenetic tree (Figure 6); the admixture analysis assigned 53.2% to the KWPN sport horse population and 33.8% to the Groninger horse population for the Groninger horse that clustered with the KWPN sport horse population in the phylogenetic tree (Figure 6). Distance between the KWPN sport horse and Groninger horse populations was indeed small (Figure 6). Also, two clusters were observed for the Gelder horse population, where one cluster was closer to the harness horse population.

## 4. Discussion

Research on the genomic makeup of horse breeds with varying stages of development and background can teach us about their breeding history and give valuable information on how to optimally conserve or improve breeds for the future. For that purpose, horses were sampled from 9 Dutch or common populations in the Netherlands that varied in stage of development and background, and genetic diversity and relationships within and among these populations were quantified using genome-wide SNP genotype data.

### 4.1. Population Specific Characteristics

#### 4.1.1. Inbreeding

In general, inbreeding in populations that allow gene-flow from other populations was lower compared to inbreeding in closed populations. Mainly gene-flow ensures genetic diversity in inbred populations of (effectively) small size [24], two properties that apply to most of the investigated populations. Based on genotype data from 17 short tandem repeats, the Friesian horse population was also found to be the most inbred population out of 35 horse populations investigated by van de Goor and colleagues [11]. Inbreeding of Friesian horses was previously stated to be primarily due to genetic drift from small effective population size during several generations since the foundation of the breed [8]. Unequal founder contribution and genetic bottlenecks also contributed to inbreeding [8]. In our study, considerable inbreeding coefficients were also obtained for Belgian draft horses and Shetland ponies, comparable to Petersen and colleagues [25] and van de Goor and colleagues [11]. Based on pedigree information, estimated inbreeding in harness horses increased with 1.36% per generation [9]. Gene-flow from other populations (e.g., Hackney or Saddlebred horses) is still performed, but not always executed optimally with respect to conservation, as a well performing outbred stallion is often mated to many dams resulting in increased kinship in subsequent generations [9].

#### 4.1.2. Runs of Homozygosity

The number and length of ROHs are in agreement with the known demography of the populations. Population bottlenecks seriously increased the number of ROHs identified within a population. The Friesian horse population stood out in mean number of ROHs. This is in line with the breeding history of Friesian horses [8], showing several periods during which the breeding population was much smaller compared to the current active breeding population [8,26,27].

Populations that allow gene-flow from other populations had less and shorter ROHs (e.g., the Gelder horse and KWPN sport horse populations), as opposed to populations with a more closed status (e.g., the Friesian horse population), similar to Metzger and colleagues [28]. As expected, the Dutch warmblood populations had a low percentage of ROH coverage (Figure 2 and Figure 3 and Table 2), except for the harness horse population. Gene-flow from other populations is allowed; harness horses are crossed with Hackney horses and American Saddlebred horses. Gene-flow would result in a breaking down of ROHs, reducing in particular the length of ROHs. However, this is not the case in harness horses as the mean length of ROHs is the largest in this population compared to all other investigated populations. The harness horse population developed from using a small set of founding horses from the Gelder horse population, with strong selection for quite a different conformation and highly specialized trot (directional inbreeding) in only a very few generations [7]. The population has a small effective size and a few elite stallions, including several outbreeds, sired many offspring (unbalanced use of stallions in breeding) [9]. As a consequence, inbreeding in the harness horse population is relatively high, and many ROHs and especially long ROHs are observed, which agrees with the findings of Schurink and colleagues [9].

Long ROHs were responsible for the larger part of genome coverage by ROHs in the Icelandic horse, Belgian draft, harness horse and Shetland pony populations, in line with a relative recent change in the breeding program of those populations, representing recent inbreeding. Based on the formula of Keller and Waller [29], the change could be 4 to 5 generations back. This time period coincides with the loss of function of horses in agriculture and the concurrent recent bottleneck due to a loss of interest in horses [6]. Despite the reasonable impact of long ROHs in the ancient Icelandic horse population, the total number of ROHs, the mean length of ROHs and the inbreeding coefficients were lowest. This possibly represents inbreeding in the past or limited founder relatedness, and inbreeding was probably built up over a long period of time (i.e. low inbreeding rate). Petersen and colleagues [25] suggested that a large census population size contributed to the high level of genetic diversity that is still present within this population, despite bottlenecks caused by isolation of the breed for several hundreds of years and natural disasters like fluorine poisoning due to a volcano eruption in 1783 [5].

A large part of the genome from Friesian horses is covered by ROHs and on average more than 200 Mb is covered by long ROHs. This corresponds with the fact that the Friesian horse population is a unique breed, largely isolated from the other populations and considerably inbred. High selective pressure for conformation traits might potentially have caused reasonable coverage of the genome by long ROHs in the other populations (Shetland pony, harness horse and Belgian draft horse population). Similarly, Metzger and colleagues [28] observed long ROHs and consequently high inbreeding in Sorraia, Thoroughbred and Arabian horses, originating from a closed population, a population with relatively narrow genetic base and/or a population experiencing high selective pressure for specific traits.

ROH statistics (number and length) per population depended on window size. E.g. the number of ROHs identified and the estimated inbreeding coefficient based on ROHs decreased when a larger window size was considered. Window size and other criteria set for a segment to be called an ROH should be considered when comparing results from the present study with literature. Moreover, it must be notified that ROHs were identified using SNP arrays of medium density. As a consequence, the amount of ROHs of small size might be underestimated. Indeed, many more ROHs (also of small size) were identified in horses from several populations by Metzger and colleagues [28] and Druml and colleagues [30], where SNP density was much higher as they applied whole genome sequencing [28] or used the HD genotyping array [30] containing about 13x more SNPs compared to the array we used. Also, Purfield and colleagues [31] compared the number of ROHs identified with the Illumina Bovine50 Beadchip in cattle to the number of ROHs identified with the BovineHD BeadChip. The medium density array was able to find 27.7% of the ROHs of 1 to 5 Mb in size that were identified with the high density array. However, almost all ROHs larger than 5 Mb in size were also identified with the medium density array. In the end, a direct comparison of the horse populations investigated within the present study is possible, as SNP density was equal and the exact same window size and criteria to call an ROH were set.

Interesting to notice is that the inbreeding coefficient based on ROHs in the warmblood populations often was higher compared to their inbreeding coefficient based on expected and observed homozygosity, while the inbreeding coefficient based on ROHs in the coldblood populations tended to be lower compared to their inbreeding coefficient based on expected and observed homozygosity. Also, more and longer ROHs were generally observed in the coldblood populations. Ascertainment bias might have added to these observations, as especially warmblood populations contributed to the development of the SNP arrays and therefore are likely to exhibit more heterozygosity compared to populations that did not contribute [32]. However, most of these warmblood populations allow gene-flow from other breeds, likely resulting in less inbreeding. We therefore cannot conclude if and to what extend ascertainment bias might have affected our results. However, inbreeding coefficient based on ROHs are more robust to ascertainment bias, as heterozygosity across the genome is affected by ascertainment bias, but long stretches of homozygous genotypes (ROHs) are less likely to occur [33]. If ascertainment bias affected our results to a greater or lesser extent, actual contrasts between the populations might be smaller.

#### 4.1.3. Linkage Disequilibrium

LD within the populations under study was moderate, like in cattle [34] and between dogs and humans [35]. The decay of LD observed in our study was similar to the decay found by Wade and colleagues [35] and Petersen and colleagues [25]. LD in the Friesian horse population was highest and comparable to the Thoroughbred horse population and various dog breeds [25,35]. High LD within these populations was expected, because breeding is performed within closed populations for over a century and the populations are derived from relatively few founders. High LD and the presence of long haplotypes within a population increases the chance of detecting an association between the genotype and phenotype (e.g., a disease) [36]. When LD is high, it is more likely that SNPs capture or tag the causal mutations. The statistical power of an association study in populations with high LD is therefore higher compared to populations with lower LD (and shorter haplotypes) when the same density of SNP array is used, e.g. [36].

Faster LD decay was observed in Icelandic horses, a more ancient population. Similarly, Wade and colleagues [35] found somewhat faster LD decay in ancient populations like the Norwegian Fjord horse population and Hokkaido population. LD across all populations was only slightly lower than the within population values. According to Wade and colleagues [35] this reflects the absence of strong bottlenecks during the formation of breeds and populations and also the need for many mares to maintain a population (as the number of offspring per mare is limited).

### 4.2. Relationships Among Populations

The quantified relationships among populations were mostly as expected based on the (breeding) history and classifications of the populations. Moreover, the relationships quantified using the MDS plot and F_ST_ values, and visualized using the admixture analysis and the phylogenetic tree were rather similar. Genetically least distant from the Friesian horses were the Belgian draft horses, the other coldblood horse population that was sampled. Identical findings were obtained by van de Goor and colleagues [11]. Also, similar to our results, Juras and colleagues [37], Petersen and colleagues [25], and van de Goor and colleagues [11] observed clustering of Shetland ponies and Icelandic horses, likely because the Shetland pony and Icelandic horse populations shared ancestors back in time [5].

The genetic distances between the Dutch warmblood populations (as presented in Figure A5) confirmed the origin of these populations. Both Groninger and Gelder horses were used for agricultural purposes several decades ago [7]. Their conformation correlated with the soil type most prevalent in the areas where the populations were formed. A somewhat heavier horse was needed for working on mostly clay soils in the province of Groningen, whereas a lighter horse was sufficient for working on more sandy soils in the province of Gelderland [7]; provinces in the Netherlands about 200 km apart. Gelder horses were introgressed with Hackney horses and American Saddlebred horses to breed harness horses with specific movement and posture. Gelder horses were also introgressed with Thoroughbred horses and stallions from Germany and France to breed sport horses with improved (sport) performance [38]. Gelder horses, harness horses and sport horses constitute the KWPN horse population [39], where admixture is performed between the different populations and observed based on the genome-wide SNP genotype data. Groninger horses are preserved and registered by the Groninger horse association as a separate warmblood population [40]. Gene-flow of the Dutch warmblood populations to the Groninger horse population occurs, although the breeding goal is focused on a heavy draft and riding horse [41]. The Lipizzaner horse population, the only non-Dutch warmblood population included in our study [12], was genetically most similar to the heaviest type of Dutch warmblood population: The Groninger horse population. Likewise, Lipizzaner horses clustered together with Groninger horses based on short tandem repeat loci in the study by van de Goor and colleagues [11]. In that same study, Lipizzaner horses were, despite their origin from Spanish horses, genetically more distant from the Iberian populations (Andalusian and Lusitano horses) compared to the Groninger horses [11]. Sharing of some ancestors in a more distant past seems therefore possible, for instance baroque horses from Germany.

### 4.3. Data Collection

Most of the samples used in our research were gathered with focus on genetic diversity research. To obtain a representative sample of a population and reliable estimation of genetic diversity, (close) relationships among individuals should be avoided as much as possible and a sufficient number of individuals per population should be sampled [42,43]. About 20 horses per population were sampled to obtain a balanced and sufficiently large dataset. However, the pairwise IBD analysis within each population showed that a few (close) relatives were present within almost all the populations. Potential effects of incorporating (close) relatives in genetic diversity research (e.g., overestimating relatedness within a population) were therefore consistent and still allowed for comparison between the populations. We considered the removal of (close) relatives, but decided not to do so as it would have considerably reduced the sample size of each population.

## 5. Conclusions and Implications

The identified genomic makeup of the native Dutch and common horse populations sampled in the Netherlands were mostly according to their historical development (as far as we know) and breeding practices. Development and breeding goals among some of these populations are found to be quite different, likely resulting in selection signatures. Future research will focus on selection signatures and regions on the genome harboring these signatures, as population differences in ROH distribution across the chromosomes were identified (data not shown), potentially identifying genes or mutations affecting particular traits within or among populations.

Clear differences were observed between the populations in inbreeding coefficients based on ROHs and the contribution of ROHs of various sizes to inbreeding, teaching us about differences in development and breeding history. In general, recent bottlenecks caused a larger contribution of long ROHs to inbreeding. Although this was also the case in Icelandic horses, likely indicating a recent and reasonable increase in inbreeding, observed inbreeding coefficients were lowest within this population and LD shortest. The absence of a strong bottleneck during the formation of this ancient population (i.e. many founders still contribute to the breed), as put forward by Wade and colleagues [35], seems a legitimate clarification as many of the other investigated populations in the Netherlands recently originated through a bottleneck usually with a high selection pressure on conformation traits.

Conserving genetic diversity within a population is important, as it among other things allows for desired or even necessary changes in the breeding goal and contributes to keeping a healthy population [23]. Through conserving genetic diversity, inbreeding is often kept to a minimum, limiting its consequences such as an increased risk for heritable disorders. However, despite inbreeding, some horse populations do not seem to experience inbreeding related problems (or are these problems not reported?) whereas other populations do. In for instance the quite extensively investigated and inbred Friesian horse population, heritable disorders such as dwarfism and hydrocephalus arose due to inbreeding [44,45], as well as inbreeding depression (e.g., reduced fertility [8]).

Populations that allow gene-flow from other populations showed less inbreeding and homozygosity. It might be challenging to identify a suitable population(s) to be used for gene-flow, although genetically comparable populations do exist [46]. Gene-flow should be conducted properly, as it can result in inbreeding as likely is observed in the harness horse population.

Maintaining genetic diversity is crucial for a healthy continuation of the investigated horse populations, that are mostly inbred and of (effectively) small size. Through tailor-made breeding practices within these populations the risk of heritable disorders can be kept to a minimum, and loss of traits and inbreeding depression can be prevented. Regular monitoring of developments within a population, such as inbreeding and number of stallions and mares used for breeding, should be performed to be able to intervene in due time.

## Figures and Tables

**Table 1:** Data description including the number of horses (n), country of origin, type (cold-, warmblood or pony), breeding (open or closed population with potential gene-flow) and a description of the 9 populations sampled in the Netherlands.

Population	N	Country of origin	Type	Breeding	Description
Belgian draft horse	23	Belgium	Coldblood	Closed	Heavy draft horse
Friesian horse	20	The Netherlands	Coldblood	Closed	Harness and riding horse
Gelder horse	20	The Netherlands	Warmblood	Open, gene-flow from warmblood horses	Light draft and riding horse
Groninger horse	20	The Netherlands	Warmblood	Open	Heavy draft and riding horse
Harness horse	20	The Netherlands	Warmblood	Open, gene-flow from Hackney and Saddlebred horses	Harness horse
Icelandic horse	20	Iceland	Pony	Closed	Gaited riding horse
KWPN sport horse	18	The Netherlands	Warmblood	Open, gene-flow from Thoroughbred and warmblood sport horses	Sport horse, jumping or dressage
Lipizzaner horse	23	Lipica, modern-day Slovenia	Warmblood	Closed, use of several sire and dam lines	Riding horse, Spanish riding school
Shetland pony	20	Shetland Isles, Scotland	Pony	Closed, four categories based on withers height	Harness and riding pony

**Table 2:** Mean, standard deviation (SD), minimum and maximum inbreeding coefficient estimated using expected and observed homozygosity ($f_{i}$) and based on runs of homozygosity ($f_{i,ROH}$) per population. Total number of ROHs identified per population, including mean, standard deviation (SD), minimum and maximum number of ROHs per horse per population is presented as well as mean, standard deviation (SD), minimum and maximum length of ROHs per population.

					Runs of Homozygosity (ROHs)												
	Inbreeding Coefficient $(f_{i}),\;\%$				Inbreeding Coefficient $(f_{i,ROH}),\;\%$				Number of ROH					Length of ROH, Mb			
Population	Mean	SD	Min	Max	Mean	SD	Min	Max	Total	Mean	SD	Min	Max	Mean	SD	Min	Max
Belgian draft horse	10.3	3.3	4.8	18.8	10.1	3.1	6.3	18.8	836	36.3	6.8	25	53	6.2	5.5	1.6	39.9
Friesian horse	25.5	3.7	18.9	33.5	22.3	4.5	9.3	30.2	1485	74.3	9.5	51	89	6.7	5.4	1.7	47.5
Gelder horse	3.1	4.3	-3.7	13.9	5.9	2.8	2.1	12.3	443	22.2	7.0	13	39	6.0	5.1	1.9	55.4
Groninger horse	2.9	3.0	-3.9	9.2	6.2	2.3	2.9	12.3	509	25.5	7.7	14	46	5.5	4.0	1.7	33.1
Harness horse	8.4	5.0	1.6	18.4	9.7	4.1	5.2	16.9	560	28.0	8.9	13	44	7.8	6.8	1.7	52.8
Icelandic horse	5.9	2.9	1.5	10.2	4.1	2.7	0.6	9.4	302	15.1	6.7	5	28	6.1	6.0	1.7	56.4
KWPN sport horse	-1.5	3.2	-6.0	5.5	5.3	2.4	1.6	11.3	400	22.2	6.7	10	34	5.4	4.2	1.9	32.8
Lipizzaner horse	6.8	2.5	2.8	12.7	9.0	2.5	4.9	14.1	729	31.7	5.6	22	43	6.4	4.9	1.9	48.2
Shetland pony	17.4	6.9	8.7	36.4	14.4	6.6	6.6	33.1	906	45.3	8.9	29	63	7.2	7.7	1.9	91.6
All 184 horses	8.8	8.6	-6.0	36.4	9.7	6.4	0.6	33.1	6170	33.5	18.1	5	89	6.5	5.8	1.6	91.6

## Appendix B

**Table A1:** Mean, standard deviation (SD), minimum and maximum inbreeding coefficient based on runs of homozygosity ($f_{i,ROH}$) per population using a window size of 15, 25 and 75 SNPs. Total number of ROHs identified per population, including mean, standard deviation (SD), minimum and maximum number of ROHs per horse per population is presented as well as mean, standard deviation (SD), minimum and maximum length of ROHs per population.

	**Inbreeding Coefficient** $(f_{i,ROH}),\;\%$																													
	**Window Size 15 SNPs**									**Window Size 25 SNPs**													**Window Size 75 SNPs**							
**Population**	**Mean**	**SD**	**Min**		**Max**					**Mean**			**SD**			**Min**			**Max**				**Mean**		**SD**		**Min**		**Max**	
Belgian draft horse	18.0	2.9	13.3		26.5					14.2			3.0			9.9			23.5				7.8		3.1		4.0		15.8	
Friesian horse	31.9	3.5	25.9		40.7					28.1			3.7			20.1			37.0				18.4		4.7		3.8		26.9	
Gelder horse	11.5	3.1	7.7		19.7					8.8			2.8			4.9			15.8				4.6		2.9		1.0		11.7	
Groninger horse	12.0	2.7	7.5		18.8					9.2			2.6			4.9			15.9				4.8		2.1		1.9		9.6	
Harness horse	15.5	4.0	10.6		22.6					12.6			4.1			7.4			19.9				8.2		3.7		4.0		15.3	
Icelandic horse	12.9	2.7	9.1		17.8					8.5			2.7			4.3			13.5				3.0		2.4		0.0		7.6	
KWPN sport horse	11.5	2.5	8.0		17.4					8.6			2.5			5.1			14.3				3.7		2.3		0.4		9.7	
Lipizzaner horse	15.2	2.4	11.7		20.2					12.3			2.5			8.8			17.7				7.4		2.4		3.5		12.5	
Shetland pony	23.5	6.5	15.6		41.7					19.5			6.7			11.7			38.5				11.8		6.5		4.2		30.4	
All 184 horses	16.9	7.3	7.5		41.7					13.6			7.1			4.3			38.5				7.8		5.7		0.0		30.4	
	**Number of ROH**																													
	**Window Size 15 SNPs**							**Window Size 25 SNPs**														**Window Size 75 SNPs**								
**Population**	**Total**	**Mean**	**SD**	**Min**		**Max**		**Total**			**Mean**			**SD**			**Min**			**Max**		**Total**		**Mean**		**SD**		**Min**		**Max**
Belgian draft horse	3401	147.9	12.0	130		169		2010			87.4			8.8			72			108		473		20.6		5.9		13		40
Friesian horse	3884	194.2	11.1	181		224		2833			141.7			11.3			124			165		957		48.9		10.0		17		65
Gelder horse	2071	103.6	14.0	81		143		1170			58.5			9.0			40			82		268		13.4		7.8		4		33
Groninger horse	2160	108.0	13.9	78		138		1261			63.1			12.4			38			92		298		14.9		5.6		7		28
Harness horse	2212	110.6	8.6	84		118		1290			64.5			10.7			43			77		370		18.5		6.8		9		32
Icelandic horse	2958	147.9	8.6	129		169		1506			75.3			8.8			57			94		142		7.1		4.6		0		16
KWPN sport horse	1979	109.9	11.3	83		130		1160			64.4			9.1			43			79		206		11.4		5.1		2		23
Lipizzaner horse	2725	118.5	8.1	105		134		1672			72.7			7.9			55			89		480		20.9		5.4		11		30
Shetland pony	3417	170.9	12.1	151		204		2184			109.2			11.6			94			136		551		27.6		8.1		16		45
All 184 horses	24,807	134.8	32.1	78		224		15,086			82.0			27.4			38			165		3745		20.4		13.0		0		65
	**Length of ROH, Mb**																													
	**Window Size 15 SNPs**								**Window Size 25 SNPs**														**Window Size 75 SNPs**							
**Population**	**Mean**	**SD**	**Min**		**Max**					**Mean**		**SD**			**Min**			**Max**					**Mean**		**SD**		**Min**		**Max**	
Belgian draft horse	2.7	3.5	1.0		40.2					3.6		4.3			1.0			40.1					8.5		6.2		3.1		39.8	
Friesian horse	3.7	4.3	1.0		47.7					4.5		4.7			1.0			47.6					8.6		5.7		3.0		46.1	
Gelder horse	2.5	3.1	1.0		55.8					3.4		3.8			1.0			55.7					7.8		5.8		2.9		55.3	
Groninger horse	2.5	2.7	1.0		33.2					3.3		3.2			1.0			33.2					7.2		4.3		3.1		33.1	
Harness horse	3.1	4.5	1.0		52.9					4.4		5.4			1.0			52.8					10.0		7.3		3.1		52.7	
Icelandic horse	2.0	2.5	1.0		56.8					2.5		3.3			1.0			56.6					9.4		7.3		3.3		56.3	
KWPN sport horse	2.3	2.6	1.0		32.9					3.0		3.1			1.0			32.9					7.3		4.9		3.3		32.8	
Lipizzaner horse	2.9	3.5	1.0		48.4					3.8		4.1			1.0			48.3					7.9		5.3		3.2		48.1	
Shetland pony	3.1	4.8	1.0		92.0					4.0		5.7			1.0			91.8					9.6		8.9		3.0		91.3	
All 184 horses	2.8	3.7	1.0		92.0					3.7		4.5			1.0			91.8					8.6		6.4		2.9		91.3	
